# Multifaceted Roles of Guanylate-Binding Proteins in Cancer

**DOI:** 10.3390/ijms26125477

**Published:** 2025-06-07

**Authors:** Derin Ahmetoglu, Haoyi Zheng, Aaron Swart, Hua Zhu, Ming Li

**Affiliations:** 1 Brain Tumor Research Centre of Excellence, Peninsula Medical School, University of Plymouth, Plymouth PL6 8BU, UK; derin.ahmetoglu@plymouth.ac.uk; 2 Department of Neurosurgery, University of Minnesota, Minneapolis, MN 55455, USA; zhen0632@umn.edu (H.Z.); aarons19377@gmail.com (A.S.); 3 Department of Pediatrics, The First Hospital of China Medical University, Shenyang 110001, China

**Keywords:** guanylate-binding proteins, GTPase, cancer, immunity

## Abstract

Guanylate-binding proteins (GBPs), encompassing GBP1 through GBP7 in humans, are interferon-inducible large GTPases of the dynamin superfamily, renowned for their pivotal roles in cell-autonomous immunity against intracellular pathogens such as viruses, bacteria, and protozoa. By recognizing pathogen-associated molecular patterns (PAMPs) and danger-associated molecular patterns (DAMPs), GBPs orchestrate lysosomal targeting, regulate inflammatory cascades, and modulate apoptosis to protect host tissues from immune-mediated damage. Beyond their foundational roles in immunity, GBPs exhibit context-dependent effects in human cancer, promoting malignancy in some tumors through enhanced immune signaling, inhibition of apoptosis, and resistance to therapies, or suppressing tumor growth through immune activation and cell cycle regulation. This comprehensive review explores the structural intricacies, immune functions, and multifaceted contributions of human GBPs to cancer, delving into their molecular mechanisms, prognostic potential, and therapeutic implications. We incorporate the latest insights to highlight how understanding GBP regulation could reshape cancer treatment strategies.

## 1. Structure, Function, and Roles of GBPs in Immunity

**Figure 1:**
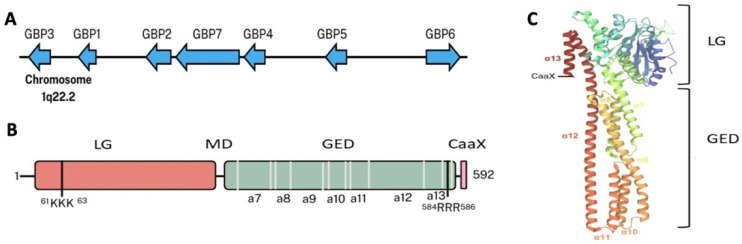
Genomic organization and structure of guanylate-binding proteins (GBPs). (**A**) Chromosomal organization of genes encoding human GBPs (hGBPs) on chromosome 1q22.2. (**B**) hGBP1 comprises an N-terminal large GTPase (LG) domain, a middle domain (MD), and a C-terminal GTPase effector domain (GED). In addition, hGBP1 contains a conserved CaaX motif essential for post-translational modification. Two positively charged stretches (^61^KKK^63^ and ^584^RRR^586^) facilitate electrostatic interactions with bacterial lipopolysaccharide (LPS). (**C**) Crystal structure of hGBP1 (PDB:1DG3). Note: This figure is adapted from Ref. [4].

Guanylate-binding proteins (GBPs) in humans, numbered GBP1 through GBP7, are large GTPases ranging from 65 to 73 kilodaltons, classified within the dynamin superfamily due to their shared ability to hydrolyze GTP and influence membrane dynamics [1,2,3]. Structurally, each GBP is a tripartite protein with distinct domains that underpin its functional versatility, including a large N-terminal GTPase (LG) domain, middle domain (MD), and a C-terminal GTPase effector domain (GED) (Figure 1). The LG domain, the catalytic core, binds and hydrolyzes GTP via a long helical “spine” in its tertiary structure, enabling cleavage to GMP in GBP1 and GBP3; but only to GDP in GBP2 and GBP5, reflecting subtle enzymatic adaptations [2,4]. This domain’s conformational flexibility allows GBPs to engage diverse substrates, from nucleotides to protein partners, making it a critical hub for regulatory modifications and effector functions. The MD, a predominantly alpha-helical segment, loops back along the LG domain, acting as a structural scaffold that ensures protein stability without direct enzymatic roles, while still facilitating interactions with cytoskeletal elements or immune signaling complexes [1,2]. Its role, though less prominent, is essential for maintaining GBP integrity under cellular stress, such as during interferon-driven activation or pathogen encounters. The GED forms a bulb-shaped cluster of helices, serving as the functional output by mediating interactions with target molecules, such as pathogen membranes, host proteins, or vacuolar structures [1,2]. In GBP1, GBP2, and GBP5, the GED includes a CaaX prenylation motif, a lipid-binding unit that enhances membrane affinity, enabling attachment to pathogen membranes or host organelles like lysosomes [1,5]. Encoded as a single gene cluster on chromosome 1q22.2 (Figure 1A), the GBP genes share 54–88% amino acid sequence identity, a testament to their conserved architecture, with high-resolution structural data derived from X-ray crystallography, nuclear magnetic resonance (NMR), and computational modeling, providing a robust foundation for mechanistic studies [1,2,3].

GBPs are tightly regulated by interferons, with interferon-gamma serving as a key inducer that rapidly upregulates their expression, triggering a response that is sustained for up to 24 h post-stimulation [1,2,6,7]. As crucial components of innate immunity, GBPs play a central role in detecting and neutralizing intracellular pathogens—including viruses, bacteria, and protozoa—by recognizing pathogen-associated molecular patterns (PAMPs) such as lipopolysaccharides (LPS) and viral glycoproteins, as well as damage-associated molecular patterns (DAMPs) like misfolded proteins and cellular debris, which signal infection or cellular stress [7].

GBP1 targets Gram-negative bacteria by binding LPS on their outer membranes, forming oligomeric complexes that recruit lysosomes to engulf and degrade invaders, a process involving homo- and heterodimerization stabilized by the GED to amplify effector functions [1,2,6,8]. GBP2 and GBP5 counter viral replication by binding to Furin, a host protease essential for processing viral glycoproteins. By blocking Furin’s activity, they prevent the integration of viral glycoproteins into the host machinery, a crucial mechanism for antiviral defense [9]. GBP3, uniquely, directly antagonizes viral RNA, offering a complementary antiviral strategy that bypasses Furin, enhancing host protection [9]. GBP4 and GBP6 reinforce the immune response by supporting GBP1 and GBP2, localizing to pathogen surfaces to enhance their activity. Meanwhile, GBP7, though less studied, is thought to play a role in restricting early viral replication, though its precise function remains unclear [2,10,11].

Beyond pathogen clearance, GBPs regulate cytoskeletal trafficking, guiding immune vesicles or organelles to infection sites—a dynamin-like trait that mirrors their membrane-remodeling cousins and is essential for immune synapse formation and pathogen containment [2,6]. This cytoskeletal role involves interactions with actin, tubulin, and integrin networks, facilitated by the GED’s prenylation motif, ensuring precise immune cell responses. GBPs play a crucial role in regulating inflammation, carefully balancing tissue protection with effective pathogen elimination. During infection or injury, GBP1 reduces endothelial cell proliferation by downregulating matrix metalloproteinase 1 (MMP1) and upregulating integrin-alpha 4, stabilizing tissue barriers and preventing excessive breakdown—a critical anti-inflammatory mechanism [12,13]. GBP5 amplifies inflammasome-driven pyroptosis via caspase-1 and caspase-4, enhancing inflammatory cell death to clear pathogens or aberrant cells, playing an integral role in defense against intracellular threats [14]. GBP2 regulates cytokine pathways and gene repair, tempering inflammatory cascades to prevent immune overactivation, while GBP3 plays an auxiliary role in caspase-4-mediated apoptosis, contributing to controlled cell death during infection [1,4]. This dual regulation, driven by interferon-gamma and cytokine networks, positions GBPs as versatile immune sentinels, bridging innate and adaptive responses in human biology.

GBPs are among the immune system’s first responders, rapidly upregulated within hours of interferon stimulation and sustained for up to 24 h. This early activation serves as a critical stopgap before slower-acting interferon-stimulated genes (ISGs) take effect [1]. Their structural conservation and precise temporal dynamics contribute to broad cellular impacts, extending beyond immunity into cancer biology, where their roles in tumorigenesis—whether oncogenic or tumor-suppressive—are intricately complex.

## 2. GBPs in Cancer: Context-Dependent Roles in Tumor Progression

The involvement of human GBPs in cancer is a labyrinth of complexity, marked by their capacity to either promote or suppress tumor progression depending on cancer type, tissue context, and microenvironmental cues—a duality summarized in Table 1. GBP1, the most comprehensively studied, exemplifies this paradox. In cancers such as renal, lung, ovarian, and glioblastoma, elevated GBP1 expression correlates with aggressive growth, metastasis, and resistance to therapies, marking it as an unfavorable prognostic factor [15,16,17,18,19,20,21]. In glioblastoma, GBP1 acts as an EGFR-induced effector, enhancing tumor growth in vivo—an effect absent in vitro, suggesting reliance on stromal or immune interactions within the brain tumor microenvironment (TME) [17]. This pro-tumor role involves GBP1’s association with EGFRvIII, a constitutively active mutant, driving actin cytoskeleton remodeling and extracellular matrix degradation to facilitate tumor spread. In lung adenocarcinoma, GBP1 boosts cell motility and invasiveness by binding beta-tubulin, increasing metastatic potential to distant sites such as lymph nodes, bones, or the brain, a process linked to its plasma membrane localization and GTPase activity [18]. In ovarian cancer, GBP1 protects cells from paclitaxel by associating with beta-tubulin and indoleamine 2,3-dioxygenase 1 (IDO-1), enhancing survival under chemotherapeutic stress and enabling drug resistance [19,20,22]. Conversely, in colorectal cancer, GBP1 suppresses tumor growth, reducing proliferation and improving overall survival, possibly by enhancing immune recognition of tumor cells—a role tied to its predominant expression in gastrointestinal tissues and interferon-driven immune activation [23]. In breast cancer, GBP1 acts as a tumor suppressor and its high expression correlates with significantly improved survival, but it can also promote brain cancer metastasis in growth factor-driven breast cancers [3]. In melanoma, high GBP1 levels correlate with better outcomes, linked to heightened immune surveillance and interferon-gamma-induced T cell infiltration, reflecting its role as an immune-activated gene [24]. This tissue-specific variability underscores GBP1’s context-dependent behavior, influenced by its expression in gastrointestinal, lymphoid, endocrine, and minor neural tissues, with RNA enriched in the liver and appendix [15].

GBP2 mirrors this complexity with equal intricacy. In breast cancer, it inhibits tumor growth and metastasis, enhancing survival by regulating mitochondrial fission via dynamin-related protein 1 (Drp-1) and promoting autophagy through autophagy-related protein 2 (ATG2), making it a favorable prognostic marker—particularly valuable for triple-negative breast cancer subtypes [36,37,38]. This anti-tumor effect involves GBP2’s homodimerization and GTPase activity, which suppress PI3K/AKT/mTOR signaling, reducing cell proliferation and sensitizing tumors to paclitaxel. In melanoma, GBP2 is shown to be a favorable prognostic marker, and its overexpression can reduce tumor malignancy by inhibiting the Wnt/β-catenin pathway [41]. In colorectal cancer, GBP2 suppresses Wnt signaling, heightening sensitivity to paclitaxel and boosting survival rates, offering a therapeutic advantage for patients facing drug-resistant tumors by inhibiting beta-catenin-driven growth [35]. In contrast, in renal carcinoma, pancreatic adenocarcinoma, and glioblastoma, GBP2 overexpression promotes malignancy, often by facilitating immune evasion or Stat3-driven invasion, leading to a poorer prognosis [25,26,31,43]. In pancreatic cancer, GBP2 acts as an acidosis-related signature, enabling tumor cells to thrive in hypoxic, acidic microenvironments, thus exacerbating disease progression by enhancing cell survival and metastasis [26]. In esophageal squamous cell carcinoma, p53-induced GBP2 upregulation signals poor survival, highlighting its oncogenic potential in specific TME contexts, driven by cooperation with interferon regulatory factor 1 (IRF-1) [44]. This variability reflects GBP2’s expression in all major tissue types except ocular, with strong presence in neural, endocrine, respiratory, gastrointestinal, and lymphoid tissues, and RNA enrichment in endocrine and respiratory tissues [27].

GBP3, though less broadly prognostic, promotes glioblastoma growth when overexpressed, activating pathways such as p62-ERK1/2 to fuel proliferation and resistance, with minimal impact on other cancers’ outcomes [32,53]. Its role involves stimulating O6-methylguanine-DNA-methyltransferase (MGMT)-mediated DNA damage repair, counteracting temozolomide efficacy, and is linked to its diffuse cellular localization, lacking a CaaX motif for membrane attachment [33]. GBP4 exhibits a split personality—favoring survival in ovarian cancer by supporting immune responses through immunomodulatory factors, yet worsening renal cancer outcomes by enhancing tumor resilience, echoing GBP1’s duality [28]. Expressed in endocrine, gastrointestinal, gallbladder, kidney, female sexual, and lymphoid tissues, GBP4’s prognostic value hinges on its Golgi and plasma membrane localization, influencing TME dynamics [28]. GBP5 supports positive outcomes in endometrial, ovarian, and colorectal cancers, often through immune infiltration, but accelerates malignancy in stomach cancer via a JAK1-STAT1/GBP5/CXCL8 positive feedback loop and in glioblastoma via Src/ERK1/2/MMP3 signaling [29,30,34,46,47,48]. Its expression is generally low across most tissues but moderate in the respiratory system, liver, and kidneys, with Golgi apparatus localization supporting its role in immune checkpoint regulation [29]. GBP6 and GBP7, the least studied members, provide preliminary insights. GBP6 exhibits reduced expression in oral squamous cell carcinoma, suggesting potential as a diagnostic marker, though findings are limited by small sample sizes [49,50]. Meanwhile, low expression of GBP6/7 levels in head and neck squamous cell carcinoma (HNSCC) correlates with shorter survival, indicating possible tumor-suppressing activity in specific contexts, while its cytoplasmic vesicle localization hints at roles in immune regulation or vesicular trafficking [51,52].

This spectrum of effects arises from GBPs’ tissue-specific expression, interactions with the TME—immune cells (e.g., tumor-associated macrophages, T lymphocytes), stromal fibroblasts, cytokines (e.g., interferon-gamma, TNF-α), and co-activated pathways (e.g., EGFR, Wnt, Stat3)—and their interferon-driven regulation [3,54]. For instance, GBP1’s pro-metastatic role in breast cancer involves T lymphocytes facilitating brain metastasis, contrasting its anti-proliferative effects in colorectal cancer, driven by differential TME composition and cytokine profiles [39,40]. GBP2’s anti-tumor effects in breast cancer hinge on mitochondrial dynamics and autophagy, while its pro-tumor role in glioblastoma leverages fibronectin remodeling and Stat3 activation, reflecting TME-specific signaling [31,36]. This variability necessitates a detailed understanding of cancer type, TME dynamics, and GBP expression patterns to predict their tumor-modulating roles accurately, a challenge that defines their study in human oncology.

### 2.1. Molecular Mechanisms of GBPs in Tumorigenesis and Therapy Resistance

GBPs influence tumorigenesis and therapy resistance through three principal mechanisms—immune modulation, treatment resistance via apoptosis and DNA repair, and metastasis via cytoskeletal dynamics—aligned with the “GBP-TME Interaction Model.” These mechanisms reveal GBPs as intricate architects of cancer biology, integrating immunity, cellular survival, and physical tumor behavior in a context-dependent manner.

#### 2.1.1. Immune Modulation

GBPs shape the TME’s immune landscape with remarkable plasticity, toggling between anti-tumor and pro-tumor effects. GBP1, rapidly upregulated by interferon-gamma within 24 h, acts as an EGFR effector in glioblastoma, promoting tumor growth and invasion through actin cytoskeleton remodeling and extracellular matrix degradation, with potential contributions to immune evasion influenced by stromal and cytokine signaling in the TME [16,17]. This evasion involves GBP1’s association with EGFRvIII, reducing antigen presentation and T cell recognition, while its plasma membrane localization facilitates immune suppression via IDO-1 interactions [55,56,57]. In colorectal cancer, GBP1 promotes immune activation by recruiting effector cells, including macrophages, dendritic cells, and T lymphocytes, to suppress tumors. This process may be mediated through LPS-like recognition of tumor debris or danger signals, influenced by its gastrointestinal expression and interferon-driven responses [23]. In pancreatic ductal carcinoma (PDAC), GBP4 is highly expressed due to its DNA hypomethylation. GBP4 overexpression promotes the infiltration of exhausted CD8+ T cells and tumor progression [58]. GBP5 increases lung cancer infiltration of B cells, CD4+ and CD8+ T cells, and NK cells, potentially sensitizing tumors to PD-L1 blockade or other immunotherapies, mediated by its Golgi localization and interferon-driven immune checkpoint modulation [59]. In ovarian cancer, GBP5 mutations impair immune efficacy, tilting the balance toward tumor escape by reducing T cell function and increasing PD-L1 expression [30]. In non-small cell lung cancer (NSCLC), GBP5 is upregulated in tumor tissues and is associated with a favorable prognosis. Its overexpression shows a strong correlation with the expression of numerous immune-related genes, including PD-L1, underscoring its potential relevance to anti-PD-1/PD-L1 immune checkpoint therapy [60]. GBP2 promotes breast cancer autophagy via ATG2, inhibiting PI3K/AKT/mTOR signaling and boosting immune sensitivity; while in osteosarcoma, it encourages immune cell infiltration (e.g., CD8+ T cells) to curb growth, driven by its homodimerization and cytokine regulation [38,61]. GBP4 enhances melanoma immune checkpoint responses, reflecting TME dynamics and aiding anti-EGFR therapy in non-small cell lung cancer, linked to its Golgi and plasma membrane localization [62,63]. These divergent outcomes (Summarized in Table 2) reflect GBPs’ ability to recalibrate immune balance, driven by interferon signaling, cytokine gradients, and TME cellularity.

#### 2.1.2. Treatment Resistance

GBPs promote tumor survival by inhibiting apoptosis and strengthening DNA repair, thereby resisting chemo-, radio-, and immunotherapies. GBP1 protects ovarian cancer cells from paclitaxel by associating with beta-tubulin, altering cytoskeletal dynamics to sequester the drug within the microtubule network, and partnering with IDO-1 to reduce apoptosis—a dual shield against cell death that enhances chemotherapeutic resistance [19,22,55]. This resistance involves GBP1’s GTPase activity stabilizing microtubules, preventing paclitaxel-induced mitotic arrest, and its IDO-1 interaction suppressing immune-mediated cytotoxicity. In lung cancer, GBP1 overexpression, spurred by circular RNA Circ_0058608, drives erlotinib resistance via PGK1-activated epithelial-to-mesenchymal transition (EMT), enabling cells to adopt a mesenchymal, drug-resistant phenotype that evades tyrosine kinase inhibitors, driven by GBP1’s plasma membrane and cytoskeletal localization [64]. GBP3 in glioblastoma upregulates MGMT to repair temozolomide-induced DNA damage, preserving tumor viability and counteracting alkylating agent efficacy, a process facilitated by its diffuse cytoplasmic distribution and GTPase activity [33]. GBP1 protein overexpression is associated with radioresistance in multiple cancers and is mainly regulated at the transcriptional step, and GBP1 knockdown by siRNA suppressed radioresistance in vitro and in xenotransplanted tumor tissues [65]. GBP5 fosters radioresistance in oral squamous cell carcinoma by activating NF-κB, suppressing apoptosis, and enhancing PD-L1 expression, creating an immune-suppressive shield that resists radiotherapy-induced cell death, linked to its Golgi-based signaling [47]. GBP2 in renal carcinoma resists traditional immunotherapies, possibly via Stat1 pathways that bolster cell survival under immune pressure, driven by its homodimerization and cytokine interactions [25]. These mechanisms collectively strengthen cancer cells against therapeutic attacks, creating major obstacles for conventional treatments and demanding innovative counterstrategies.

#### 2.1.3. Metastasis

GBPs regulate cell motility and invasion through cytoskeletal remodeling, either propelling or restraining metastatic spread. GBP1 enhances lung adenocarcinoma invasiveness by binding beta-tubulin, increasing cell motility, and enabling metastatic dissemination to distant organs like lymph nodes, bones, or brain, driven by its GTPase activity and plasma membrane localization [18]. This pro-invasive role involves GBP1’s association with EGFR or EGFRvIII, inducing MMP1 expression in glioblastoma [16,17]. In breast cancer, GBP1 promotes lymph node and brain metastasis with T lymphocyte support, enabling blood–brain barrier infiltration through cytoskeletal remodeling and immune interactions, driven by its granular cytoplasmic localization and interferon-induced expression [39,40]. GBP2 drives glioblastoma invasion via the Stat3/fibronectin pathway, linking immune signaling to extracellular matrix remodeling and physical tumor spread, facilitated by its homodimerization and cytoskeletal interactions [31]. Contrarily, GBP2 inhibits breast cancer metastasis by regulating Rho GTPases, limiting invadosome formation and cell migration—a protective cytoskeletal brake driven by its GTPase activity and autophagy induction [37]. GBP5 promotes glioblastoma matrix degradation via Src/ERK1/2/MMP3, aiding vascular co-option and metastatic progression, linked to its Golgi-based signaling and immune modulation [34]. GBP4 in oral cancers, when unmethylated, may drive sarcoma progression, hinting at cytoskeletal roles via integrin or actin dynamics, though mechanisms require further elucidation [66]. These cytoskeletal roles underscore GBPs’ capacity to modulate metastatic potential, contingent on tumor type, TME composition, and molecular signaling, offering a rich target for therapeutic intervention.

Together, these mechanisms reveal GBPs as multifaceted regulators of cancer biology, integrating immune responses, cellular resilience, and tumor dissemination in a context-dependent manner. Their ability to adapt to local TME conditions—immune pressure, therapeutic stress, or physical barriers—makes them both drivers of malignancy and potential vulnerabilities for targeted therapies, with profound implications for oncology.

### 2.2. GBPs as Biomarkers for Cancer Prognosis and Treatment Response

GBPs’ expression profiles, cellular localization, and immune interactions position them as potent biomarkers for prognosis and treatment response, offering actionable insights into tumor behavior and therapeutic outcomes. GBP1 exemplifies this dual utility. Its overexpression in glioblastoma, lung, ovarian, and renal cancers signals poor prognosis, reflecting increased malignancy, metastasis, and resistance to standard therapies like paclitaxel, temozolomide, or erlotinib [16,17,19,21,24]. In glioblastoma, GBP1’s EGFR-driven role marks it as a malignancy indicator, detectable near the plasma membrane or in cytoplasmic granules via immunohistochemistry, providing a reliable prognostic tool [16,17,67]. In lung cancer, elevated GBP1 is linked to erlotinib resistance, serving as a prognostic warning for EGFR-targeted therapies and detectable via quantitative PCR (qPCR), proteomic assays, or circulating tumor DNA analysis [68]. Conversely, in colorectal cancer and melanoma, elevated GBP1 predicts favorable outcomes, linked to reduced proliferation or enhanced immune surveillance, detectable in tissue biopsies, peripheral blood, or circulating tumor cells via enzyme-linked immunosorbent assay (ELISA), mass spectrometry, or immune profiling [23,24]. Pan-cancer analyses indicate that patients with higher GBP1 expression are more likely to exhibit “hot” anti-tumor immune phenotypes, characterized by lower Tumor Immune Dysfunction and Exclusion (TIDE) scores and higher immunophenoscores, suggesting a greater likelihood of responding to immunotherapy [69]. In contrast, GBP2 is downregulated in skin cutaneous melanoma and is associated with reduced immune cell infiltration and poorer prognosis. Notably, high promoter methylation of GBP2 has been proposed as a potential biomarker for unfavorable outcomes in this cancer type [42]. GBP2 serves as a positive prognostic marker in breast cancer, where it correlates with reduced tumor growth, heightened paclitaxel sensitivity, and improved survival—particularly valuable for triple-negative subtypes—detectable in blood as a minimally invasive tool via ELISA or mass spectrometry, offering a practical diagnostic approach [36,38]. In pancreatic adenocarcinoma, however, high GBP2 forecasts poor survival, tied to immune evasion and acidosis adaptation, quantifiable through RNA sequencing, tissue proteomics, or tumor microenvironment analysis [43,70].

GBP5 emerges as a pan-cancer biomarker, with overexpression linked to immune checkpoint behavior and immunotherapy response across multiple tumor types, such as lung and triple-negative breast cancer [59,71,72,73]. In lung cancer, high GBP5 indicates potential sensitivity to immune checkpoint blockers like PD-L1 inhibitors, driven by immune cell infiltration (e.g., CD8+ T cells, NK cells), measurable in respiratory tissues, bronchoalveolar lavage, or blood samples via flow cytometry, qPCR, or single-cell RNA sequencing, providing a robust predictive tool [59]. In triple-negative breast cancer, inhibiting GBP5 boosts PD-L1 efficacy, indicating its potential as a predictive marker for immunotherapy success. Its levels can be measured through apoptosis assays, Western blot analysis, or immune profiling, facilitating patient stratification [73]. GBP4 reflects TME dynamics, predicting immunotherapy responsiveness in melanoma and non-small cell lung cancer, with unmethylated forms in oral cancers serving as tumor-specific markers, detectable through methylation-specific PCR, next-generation sequencing, or epigenetic profiling, offering a niche diagnostic utility [62,63,74]. Localized to the Golgi apparatus and plasma membrane, GBP4’s expression provides microenvironmental insights, observable via immunofluorescence, tissue microarrays, or spatial transcriptomics, enhancing its prognostic value [28]. GBP3, while not broadly prognostic, exhibits glioblastoma-specific overexpression, activating p62-ERK1/2 and potentially aiding targeted diagnostics through RNA in situ hybridization, microarray analysis, or proteomic assays. However, its clinical utility needs further validation [32,53]. GBP6’s reduction in oral squamous cell carcinoma and tongue squamous cell carcinoma hints at diagnostic potential, measurable in saliva, tissue biopsies, or circulating DNA via qPCR, RNA sequencing, or liquid biopsies, pending larger cohort confirmation [50]. GBP7’s elevation in HNSCC predicts shorter survival, a niche marker assessable via transcriptomics, tissue microarrays, or immune profiling, awaiting broader validation to establish its prognostic relevance [52].

Therapeutically, GBP levels inform treatment strategies with precision. Knocking down GBP1 restores erlotinib sensitivity in lung cancer, shrinking tumors and prolonging G1 phase arrest, trackable via cell cycle analysis, positron emission tomography (PET) imaging, or tumor biopsies, offering a dynamic response marker [68]. Suppressing GBP5 boosts chemotherapy efficacy in breast cancer, enhancing taxane response and reducing immune evasion, quantifiable through apoptosis assays, immune profiling, or tumor size reduction metrics, aiding treatment monitoring [73]. GBP2’s paclitaxel sensitization in colorectal cancer suggests its upregulation could guide adjuvant therapy, monitored via drug sensitivity screens, tumor biopsies, or circulating tumor DNA analysis, optimizing therapeutic outcomes [35]. These examples underscore GBPs’ potential to personalize cancer care, though their variable effects demand tumor-specific validation, standardized detection methods (e.g., ELISA, NGS, spatial transcriptomics), and integration into clinical workflows for routine use, ensuring robust prognostic and predictive utility.

## 3. Therapeutic Targeting of GBPs: Challenges and Opportunities

Targeting GBPs offers a tantalizing therapeutic frontier, leveraging their roles in immunity, resistance, and metastasis, yet their dual nature presents formidable challenges. Inhibiting GBP1 may overcome resistance in cancers such as ovarian and lung, where sh-GBP1 inhibits tumor growth and restores erlotinib sensitivity by disrupting EMT signaling and IDO-1 interactions. This strategy can be tested in preclinical models and clinical trials [55,68]. Disrupting GBP1-IDO-1 interactions with agents like astragaloside IV diminishes immune evasion, enhancing chemo- or immunotherapy efficacy, a mechanism evaluable through immune profiling, tumor growth assays, or patient-derived xenografts [55]. Enhancing GBP2 activity in breast cancer, possibly via Drp-1 agonists, autophagy inducers, or small molecule activators targeting its GTPase domain, could suppress mitochondrial replication and tumor growth, capitalizing on its protective effects against metastasis—a strategy assessable through mitochondrial function assays, tumor size monitoring, or drug sensitivity screens [36,38]. Modulating GBP5—suppressing it in breast cancer with siRNA or enhancing it in lung cancer with agonists—could optimize immunotherapy outcomes, leveraging its immune checkpoint influence to boost PD-L1 blockade efficacy, monitorable via immune cell infiltration assays, T cell function tests, or tumor response metrics. In colorectal cancer, upregulating GBP2 could enhance the response to paclitaxel, offering a promising combinatory approach. This can be monitored through tumor biopsies, drug sensitivity screens, or circulating tumor DNA analysis [35].

Challenges, however, are legion. GBP1’s tumor-suppressive role in colorectal cancer contrasts with its oncogenic effects in glioblastoma, risking adverse outcomes with broad inhibition—suppressing it might shrink gliomas but spur colorectal tumors, a complexity necessitating tumor-specific precision [16,17,23,67]. GBP2’s beneficial effects in breast cancer contrast with its role in driving malignancy in renal carcinoma, necessitating precise targeting strategies, which can be evaluated through tumor-specific expression profiling or patient stratification [25,36]. Systemic modulation could disrupt GBPs’ immune roles, impairing pathogen defense—a concern given their interferon-driven functions in viral, bacterial, and protozoal clearance, evaluable through safety studies or immune function assays [7]. Delivery precision is critical—nanoparticle encapsulation, tumor-specific promoters, or antibody-drug conjugates might localize effects, but off-target risks to healthy tissues persist, particularly in immune-rich organs like liver or lymphoid tissues where GBPs are expressed, monitorable via pharmacokinetic studies or tissue-specific assays. Moreover, in vivo efficacy often diverges from in vitro results, as with GBP1 in glioblastoma, requiring robust preclinical models (e.g., patient-derived xenografts, organoids) to bridge the gap, assessable through comparative efficacy trials [16,17]. Resistance mechanisms—e.g., compensatory upregulation of other GBPs (e.g., GBP5 for GBP1 inhibition), alternative pathways like EGFR or Stat1 signaling, or epigenetic adaptations—could undermine single-target approaches, necessitating multi-pronged strategies, evaluable through resistance profiling or longitudinal studies [25,68].

Opportunities abound in combination therapies. Pairing GBP inhibitors with chemotherapy (e.g., paclitaxel, temozolomide) or immune checkpoint blockers (e.g., PD-L1, CTLA-4) could overcome resistance, as seen with GBP5 in breast cancer and GBP3 in glioblastoma—a strategy testable in clinical trials or preclinical models [33,53,71,73,75]. Developing selective modulators—small molecules targeting GBP1’s GTPase domain, RNA interference silencing GBP5, or CRISPR-based editing of GBP2—could refine specificity, assessable through high-throughput screens, structural biology, and patient-derived models, offering precision medicine potential. Advances in drug delivery, such as liposomal carriers targeting GBP-rich TMEs, bispecific antibodies linking GBP inhibition to immune activation, or tumor-specific nanoparticles, might enhance tumor specificity, monitorable via pharmacokinetic and pharmacodynamic studies. Preclinical success with GBP1 knockdown in lung cancer suggests translational potential, but scaling to humans requires overcoming pharmacokinetic hurdles (e.g., half-life, bioavailability), safety concerns (e.g., immunogenicity), and clinical trial design, assessable through phase I/II studies [68]. Patient stratification—using GBP expression profiles from biopsies, circulating tumor DNA, or immune profiling—could identify responders, optimizing clinical trials and therapeutic outcomes, monitorable via real-time biomarker tracking or imaging. Despite these obstacles, GBPs’ multifaceted roles offer a rich landscape for innovative cancer treatments, provided research navigates their complexity with ingenuity, rigor, and precision, leveraging advanced translational tools and clinical validation strategies.

### Future Directions and Unanswered Questions

The future of GBP research in human cancer is a horizon brimming with promise and perplexity, with a variety of questions awaiting exploration to unlock their full potential. What molecular switches—genetic polymorphisms, epigenetic modifications (e.g., methylation, histone acetylation), environmental cues (e.g., hypoxia, nutrient stress), or TME-specific factors—dictate their context-specific effects? How do TME components—immune cells (e.g., tumor-associated macrophages, T lymphocytes), stromal fibroblasts, cytokine gradients (e.g., interferon-gamma, TNF-α), biophysical factors like pH, or microbial influences—shape GBP behavior, and can these be modeled in organoids, 3D cultures, or patient-derived xenografts to predict tumor responses? Why do GBP1 and GBP2 exhibit opposing roles across cancers—e.g., tumor suppression in colorectal cancer versus oncogenicity in glioblastoma—and can machine learning, multi-omics analysis, or systems biology predict these patterns from genomic, proteomic, or transcriptomic data [16,17,23,31,36]. Unraveling these drivers could pinpoint therapeutic windows, tailoring interventions to tumor subtypes and TME profiles with precision.

What specific signaling pathways—beyond EGFR, Wnt, Stat3, or PI3K/AKT/mTOR—regulate GBP expression or function in cancer, and can these be targeted for synergy with existing therapies? For instance, does GBP1’s IDO-1 interaction extend to other immune checkpoints like CTLA-4 or LAG3, or does GBP5’s PD-L1 enhancement involve novel cytokine loops, assessable through pathway analysis or immune profiling [55,73]? How do GBPs interact with epigenetic regulators—e.g., histone deacetylases, DNA methyltransferases—driving their context-specific expression, and can epigenetic therapies like inhibitors of DNMT or HDAC enhance GBP-targeted strategies, evaluable through chromatin immunoprecipitation or methylome sequencing [74]? Are there temporal dynamics—acute versus chronic interferon exposure, or early versus late-stage TME changes—that shift GBP roles from tumor suppression to promotion, monitorable through longitudinal tumor studies or time-series transcriptomics [7]?

Technological advances offer pathways forward. Pan-cancer genomics, leveraging databases like Pan-cancer genomics, utilizing databases such as The Cancer Genome Atlas (TCGA) and the International Cancer Genome Consortium (ICGC), can map GBP expression, mutations, and splicing variants across tumor types. This mapping helps clarify prognostic patterns and identify therapeutic targets, which can be analyzed through bioinformatic pipelines or machine learning models [52]. Single-cell RNA sequencing might dissect TME-GBP interactions at granular resolution, revealing cell-specific roles—e.g., GBP5 in tumor-infiltrating lymphocytes versus cancer cells, monitorable through spatial transcriptomics or CyTOF analysis [59]. CRISPR screens could identify upstream regulators (e.g., transcription factors, miRNAs like miR-29 for GBP2) or downstream effectors, while proteomics might uncover novel GBP interactors beyond Furin, IDO-1, or beta-tubulin, assessable through mass spectrometry or protein–protein interaction networks [25]. Developing GBP-targeted drugs—e.g., GTPase inhibitors for GBP1 in glioblastoma, agonists for GBP2 in breast cancer, or allosteric modulators for GBP5—requires high-throughput screening, molecular docking, and in vivo validation in patient-derived xenografts or organoids to ensure efficacy and tolerability, monitorable through preclinical efficacy trials [36,68]. Combination strategies with existing therapies—PD-L1 inhibitors, DNA-damaging agents like cisplatin, metabolic inhibitors, or epigenetic modulators—merit rigorous testing, given GBPs’ ties to immunity, repair, cellular stress, and TME signaling, evaluable through synergy screens or clinical studies [33,73]. Longitudinal studies could assess GBPs’ impact on recurrence, metastasis, or therapy resistance over years, addressing gaps in current cross-sectional data and informing adjuvant strategies, monitorable through tumor tracking, imaging, or liquid biopsies [61].

Unanswered questions extend beyond cancer biology. How do GBPs interplay with metabolic pathways—e.g., glycolysis, lipid synthesis, or oxidative phosphorylation—in tumors, potentially influencing energy availability for growth, immune evasion, or chemotherapeutic resistance? Could their immune roles modulate microbiome-tumor interactions, given LPS recognition’s overlap with gut flora and GBP1’s gastrointestinal expression [1,23]? Are there sex- or age-specific GBP effects, driven by hormonal regulation (e.g., estrogen, testosterone) or senescence, as hinted in breast cancer subtypes or HNSCC, assessable through cohort studies or hormonal profiling [3,52]? Environmental factors—diet, smoking, viral co-infections (e.g., HPV in HNSCC), or chronic inflammation—might further modulate GBP expression, warranting epidemiological studies or environmental exposure models [52]. Significant translational challenges remain: How can GBP-targeted therapies achieve efficacy while preserving immune homeostasis, avoiding immunosuppression, and maintaining pathogen defense? These concerns can be evaluated through safety trials and immune function assays [7]. Can biomarkers like GBP4 methylation, GBP5 splicing, or GBP1 plasma levels be standardized for clinical diagnostics, integrated into routine oncology workflows, and validated through large-scale clinical trials or real-world evidence studies [62,76,77,78]. Addressing these gaps through interdisciplinary efforts—molecular biology, immunology, oncology, bioinformatics, and clinical translational science—could transform GBPs into precision medicine cornerstones. The outlook is luminous, but rigorous, collaborative investigation is essential to harness their full therapeutic and prognostic potential in cancer biology.

## 4. Conclusions

GBPs (GBP1–GBP7) are interferon-driven GTPases with conserved structures and diverse functions, from pathogen defense to cancer modulation. Their tripartite architecture—LG, MD, and GED—enables GTP hydrolysis, membrane binding, and effector interactions, underpinning immune roles in lysosomal targeting, apoptosis regulation, and cytoskeletal control. In cancer, GBPs exhibit context-dependent effects, promoting malignancy in some situations (e.g., glioblastoma, lung) while suppressing it in others (e.g., colorectal, breast), driven by immune modulation, therapy resistance, and metastatic dynamics. As biomarkers, they offer prognostic and predictive insights, guiding personalized treatment, while as therapeutic targets, they promise innovative strategies, tempered by their complexity and TME-specific challenges. Further exploration—spanning molecular mechanisms, TME interactions, and clinical translation—holds the key to unlocking GBPs’ potential, bridging immunity and oncology in transformative ways for human health.

## Figures and Tables

**Table 1:** Biological functions of GBP1–7 in various cancers.

Cancer Type	Promoting or Suppressing Cancer	Mechanisms	Ref
Renal Cancer	GBP1/GBP2/GBP4: Promoting	GBP1: Enhances growth and metastasis via EGFR signaling and actin remodeling. GBP2: Facilitates immune evasion and Stat3-driven invasion. GBP4: Enhances tumor resilience via Golgi/plasma membrane localization.	[15,25,26,27,28]
Lung Adenocarcinoma	GBP1: Promoting	Binds beta-tubulin to boost motility and metastasis to lymph nodes, bones, or brain via GTPase activity.	[18]
Ovarian Cancer	GBP1: Promoting; GBP4/GBP5: Suppressing	GBP1: Protects from paclitaxel via beta-tubulin/IDO-1, enhancing drug resistance. GBP4: Supports immune responses. GBP5: Promotes immune infiltration.	[19,21,22,28,29,30]
Glioblastoma	GBP1/GBP2/GBP3/GBP5: Promoting	GBP1: EGFR-induced effector, drives actin remodeling and matrix degradation. GBP2: Facilitates immune evasion and Stat3-driven invasion. GBP3: Activates p62-ERK1/2 and MGMT-mediated DNA repair. GBP5: Activates Src/ERK1/2/MMP3 signaling.	[16,17,31,32,33,34]
Colorectal Cancer	GBP1/GBP2/GBP5: Suppressing	GBP1: Enhances immune recognition via IFN-γ. GBP2: Inhibits Wnt signaling, enhances paclitaxel sensitivity. GBP5: Supports immune infiltration.	[23,29,35]
Breast Cancer	GBP1: Dual (Suppressing in IFN-driven, Promoting in growth factor-driven); GBP2: Suppressing	GBP1: Suppresses growth in IFN-driven cancers; promotes brain metastasis via T lymphocytes in growth factor-driven cancers. GBP2: Inhibits growth via Drp-1/ATG2, suppresses PI3K/AKT/mTOR, and sensitizes to paclitaxel.	[3,36,37,38,39,40]
Melanoma	GBP1/GBP2: Suppressing	GBP1: Enhances immune surveillance and IFN-γ-induced T cell infiltration. GBP2: Inhibits Wnt/β-catenin pathway.	[24,41,42]
Pancreatic Adenocarcinoma	GBP2: Promoting	Enhances survival and metastasis in hypoxic, acidic TME as an acidosis-related signature.	[26,43]
Esophageal Squamous Cell Carcinoma	GBP1/GBP2: Promoting	GBP1: Promotes lymph node metastasis and poor prognosis via enhanced invasion and migration. GBP2: A p53-regulated target gene to promote tumor growth.	[44,45]
Endometrial Cancer	GBP5: Suppressing	Supports immune infiltration.	[29,30,34,46,47,48]
Stomach Cancer	GBP5: Promoting	Drives JAK1-STAT1/GBP5/CXCL8 feedback loop.	[48]
Oral Squamous Cell Carcinoma	GBP6: Potential Suppressing	Reduced expression; potential diagnostic marker.	[49,50]
Head and Neck Squamous Cell Carcinoma (HNSCC)	GBP1/GBP2/GBP7: Suppressing	Low expression correlates with shorter survival; linked to immune regulation and vesicle localization.	[51,52]

**Table 2:** Summary of GBP functions in tumor-TME interactions.

GBP	Cancer Type	Function in TME Interaction	Key Mechanisms	Ref
GBP1	Colorectal Cancer	Enhances immune activation	Recruits effector cells (macrophages, dendritic cells, T lymphocytes) via LPS-like recognition of tumor debris; driven by gastrointestinal expression and IFN-γ responses.	[23]
GBP2	Breast Cancer	Boosts immune sensitivity	Promotes autophagy via ATG2, inhibiting PI3K/AKT/mTOR signaling; enhances immune cell infiltration (e.g., CD8+ T cells) through homodimerization and cytokine regulation.	[38,61]
GBP2	Osteosarcoma	Enhances immune cell infiltration	Promotes CD8+ T cell infiltration to suppress tumor growth; driven by homodimerization and cytokine regulation.	[38,61]
GBP4	Pancreatic Ductal Carcinoma (PDAC)	Promotes tumor progression	Overexpressed due to DNA hypomethylation; increases infiltration of exhausted CD8+ T cells, supporting tumor growth.	[58]
GBP4	Melanoma	Enhances immune checkpoint responses	Modulates TME dynamics to support anti-EGFR therapy; linked to Golgi and plasma membrane localization.	[62,63]
GBP4	Non-Small Cell Lung Cancer (NSCLC)	Supports anti-EGFR therapy	Enhances immune checkpoint responses; linked to Golgi and plasma membrane localization.	[62,63]
GBP5	Lung Cancer (NSCLC, SCLC)	Enhances immune cell infiltration	Increases infiltration of B cells, CD4+ and CD8+ T cells, and NK cells; sensitizes tumors to PD-L1 blockade via Golgi localization and IFN-γ-driven immune checkpoint modulation.	[59]
GBP5	Ovarian Cancer	Impairs immune efficacy	Mutations reduce T cell function and increase PD-L1 expression, promoting tumor escape.	[30]
GBP5	Non-Small Cell Lung Cancer (NSCLC)	Associated with favorable prognosis	Upregulated in tumor tissues; correlates with immune-related gene expression (e.g., PD-L1), enhancing anti-PD-1/PD-L1 therapy efficacy.	[60]
